# Commentary: Autism: A model of neurodevelopmental diversity informed by genomics

**DOI:** 10.3389/fpsyt.2023.1113592

**Published:** 2023-01-24

**Authors:** Darko Sarovic

**Affiliations:** ^1^Gillberg Neuropsychiatry Centre, Department of Psychiatry and Neurochemistry, Institute of Neuroscience and Physiology, Sahlgrenska Academy, University of Gothenburg, Gothenburg, Sweden; ^2^Harvard Medical School, Boston, MA, United States; ^3^Athinoula A. Martinos Center for Biomedical Imaging, Department of Radiology, Massachusetts General Hospital, Boston, MA, United States; ^4^Department of Radiology, Sahlgrenska University Hospital, Gothenburg, Sweden; ^5^MedTech West, Gothenburg, Sweden

**Keywords:** autism, theoretical framework, model, genetic architecture, neurodevelopmental disorder (NDD), rare genetic variant, common genetic variant

In their paper, Chawner and Owen (1) present a genetic model for autism that outlines two contributory factors: (1) a social and adaptive continuum due to common genetic variation; and (2) a neurodevelopmental continuum due to rare genetic variation that presents itself as a continuum of impairment spanning from intellectual disability, through autism and ADHD, to schizophrenia and bipolar disorder.

I applaud the authors on relating the main mechanisms of the model to the differing views between the neurodiversity community and that of the medical model regarding the nature of autism, as they pertain to different aspects of the phenotype and both being important for explaining variability in clinical presentations. The model itself is very similar to part of a more comprehensive model I previously proposed (2, 3) and although there are many similarities between the papers, it is worth noting some empirical differences with important ramifications. I will argue that their conceptualization is not supported by the current literature and that it contains an issue that limits its practical usefulness. I will conclude by presenting testable postulates arising from the two models which will allow future studies to empirically validate them.

They write that the “neurodevelopmental continuum […] results in a diverse spectrum of outcomes,” referring to individual diagnoses under the neurodevelopmental umbrella. It does that through the effects of rare genetic mutations and environmental risk factors. As they operationalize it, the magnitude of the rare genetic burden determines which phenotype develops, and ultimately which diagnosis is received [see Figure 1 in (1)]. Conceptually, greater impairment is more closely associated with intellectual disability and autism than with schizophrenia and bipolar disorder.

Although the apparent statistical associations of these features appear in the literature, there is an issue with this operationalization that can be illustrated with an example. Consider an individual with a rare genetic burden of a given magnitude (*X*_*inherited*_) and a diagnosis of bipolar disorder. If that individual were to have a child with inherited said burden, but with additional *de novo* variants (*X*_*inherited*_ + *X*_*de novo*_) that child should be more likely to develop autism or ADHD than bipolar disorder. The idea that the type of condition one develops is contingent on the magnitude of rare genetic risk is not supported by empirical evidence. The conditions have partly independent genotypic (4–7), and neuroendophenotypic signatures (8, 9), suggesting that they also have partly different biological backgrounds, rather than them being part of a single continuum. A person with bipolar disorder can certainly have a lower IQ and greater “cognitive impairment” than someone with an autism diagnosis. The operationalization of the neurodevelopmental continuum alludes to a causative mechanism by which the magnitude of the rare genetic burden impacts specificity of diagnosis. This is empirically unlikely given the state of the literature, unless the continuum is a pseudo-unidimensional manifold rather than linear, and it therefore probably represents a statistical artifact.

Furthermore, following the conceptualization of a neurodevelopmental continuum, the addition of a social-adaptive factor to the model is not without issues since the autistic phenotype (which also encompasses such traits) is already conceptualized along the first factor. Clearly, the second factor is conceptualized in order to accommodate the literature on the association between autism and common genetic variation. However, within the proposed model one cannot dissociate the autistic phenotypes residing within each of the factors (whether an autistic trait belongs to the social-adaptive or the neurodevelopmental continuum), greatly limiting the practical utility of the proposed model.

Their operationalization can be contrasted with that of the pathogenetic triad (2, 3), which previously suggested that there is (1) natural variation in non-pathological traits (such as autistic or schizotypal) due to common genetic variation, and (2) a range of neurodevelopmental risk factors including, but not limited to, rare genetic variation. These risk factors negatively influence brain and cognitive development, and limit adaptive behaviors. Notably, adaptive behavior is conceptualized within a third factor that moderates the association between the first two factors in giving rise to a diagnosis. This is an important distinction since Chawner and Owen seem to conceptualize adaptive behavior within the first factor as “social-adaptive traits” (although, they do not formally operationalize it). These two factors additively influence the risk, and crucially, the first factor provides the model with disorder specificity (through common variant burden for each condition, not rare burden). Also, rather than the magnitude of neurodevelopmental risk factors affecting which condition develops (as in their model), it non-specifically determines the probability of fulfilling criteria for any one diagnosis (or multiple).

Although the models are similar, there are subtle differences that give rise to different empirical predictions, each with testable postulates. In Table 1 present a few of these predictions, and the patterns in the existing and future literature that would favor one model or the other (some of which are already supported or undermined).

**Table 1:** Testable postulates for differences between the operationalizations.

	**Genomic neurodevelopmental model (1)**	**Pathogenetic triad (2, 3)**
Specificity of diagnosis	Which diagnosis one receives depends on the individual rare variant genomic profile. Individuals are less likely to have conditions or endo-/phenotypes that are further from each other along the neurodevelopmental continuum [e.g., unlikely co-occurrence of BD and ID; (10, 11)].	Which diagnoses one receives depends on the individual common variant genomic profile.
The presence of multiple diagnoses	Individuals should only be able to get one diagnosis (although they write “frequent co-morbidity,” one cannot be located in two positions along a single continuum and simultaneously have a high and low rare genetic burden). The main clinical difficulty lies in ascertaining between those that are close to each other along the neurodevelopmental continuum.	A higher neuropathological burden is positively associated with the risk of any one diagnosis, and the number of co-occurring diagnoses. The higher the burden, the more of the different disorder-specific traits/personality types (autistic, schizotypal etc.) become maladaptive and fulfill diagnostic criteria (12, 13).
Transgenerational inheritance pattern	Which condition(s) one develops is less related to the traits or conditions of the parents, and instead depends on the magnitude of rare genetic burden and risk factors.	The condition(s) one develops depends on which common variants, traits and conditions the parents have. Parents that have a higher magnitude of rare genetic variants and risk factors are more likely to have children with any, and multiple condition(s).
Distribution of traits in the population	[See Figure 2 in (1)] An intermediate rare and common burden do not additively give rise to an autistic-like phenotype (due to empty area of plot), implying a non-continuous distribution and a strictly non-linear additivity for common and rare variants.	[See Figure 2 in (3); Y-axis conceptually inverted compared with (1)] The first and second factor are both continuously distributed in the population (14), with additivity for common and rare variants.

BD, Bipolar Disorder; ID, Intellectual Disability.

## Author contributions

The author confirms being the sole contributor of this work and has approved it for publication.
